# Is It Possible to Produce Certified Hazelnut Plant Material in Sicily? Identification and Recovery of Nebrodi Genetic Resources, *in vitro* Establishment, and Innovative Sanitation Technique From *Apple Mosaic Virus*

**DOI:** 10.3389/fpls.2021.778142

**Published:** 2021-12-16

**Authors:** Emna Yahyaoui, Daniela Torello Marinoni, Roberto Botta, Paola Ruffa, Maria Antonietta Germanà

**Affiliations:** ^1^Dipartimento Scienze Agrarie, Alimentari e Forestali (SAAF), Università degli Studi di Palermo, Palermo, Italy; ^2^Dipartimento di Scienze Agrarie, Forestali e Alimentari (DISAFA), Università degli Studi di Torino, Turin, Italy

**Keywords:** tissue culture, micropropagation, encapsulation, SSR, DNA fingerprinting, meristem tip culture, synthetic seed, *Corylus avellana* L.

## Abstract

Eight Sicilian cultivars of hazelnut (*Corylus avellana* L.), namely-Curcia, Nociara Collica, Panottara Collica, Panottara Galati Grande, Parrinara, Panottara Baratta Piccola, Enzo, and Rossa Galvagno, registered into the Italian Cultivar Register of fruit tree species in 2017 were selected from Nebrodi area and established *in vitro*. The aim of the work was to carry out the sanitation of the cultivars and get virus-free plants from the most important viral pathogen threat, the *apple mosaic virus*. Virus-free plant material is essential for the production of certified plants from Sicilian hazelnut cultivars, complying the CE (cat. CAC) quality and the technical standards established in 2017 for voluntary certification by the Italian Ministry of Agricultural, Food and Forestry Policies (MIPAAF). In this study, we investigated the possibility of establishing *in vitro* true-to-type and virus-free hazelnut plantlets *via* the encapsulation technology of apexes. The *in vitro* shoot proliferation rates were assessed for the different cultivars, sampling periods, temperature treatments, and type of explant used for culture initiation. Viability, regrowth, and conversion rates of both conventional meristem tip culture (MTC) and not conventional (MTC combined with the encapsulation technology) sanitation techniques were evaluated.

## Introduction

Italy is the second main largest producer of hazelnut (*Corylus avellana* L.) in the world after Turkey, with 98,530 tons in-shell nuts per year. Average yield is 12,417 tons per 79,350 ha of harvested area mainly concentrated in Campania, Piemonte, Latium, and Sicily regions (Food and Agriculture Organization of the United Nations, 2019). The Italian cultivars Tonda di Giffoni and Tonda Gentile delle Langhe are the most preferred hazelnuts in the market because of their well-shaped kernels suitable for processing and the high organoleptic quality (Petriccione et al., 2010; Caligiani et al., 2014; Ciarmiello et al., 2014; Silvestri et al., 2021). Nut traits are heterogeneous in hazelnut (particularly in the Sicilian cultivars) and highly dependent on the genotype, agricultural techniques, and environmental factors (Król and Gantner, 2020). Nebrodi is one of the oldest inhabited areas in Sicily, and it is considered very favorable for wild and cultivated hazelnut growing since ancient times. Sicilian hazelnuts with lower qualities were directed to the market and sold at lower prices as an approach to overcome the poverty problem leading to uncontrolled fruit quality. As a consequence, the Sicilian hazelnut production has been permanently characterized by a high morphological variability, mainly due to heterogeneity of cultivars and lack of superior elite genotypes.

Certification programs were designed within this context for the mass production of propagated plant material free of pathogens that satisfies the recommended CE (cat. CAC) quality and technical standards recently published in 2017 by the Ministry of Agricultural, Food and Forestry Policies (MIPAAF) for the production of certified hazelnut propagated materials. Within this frame of work, we investigated the production of prebasic plant material to guarantee the phytosanitary and true-to-type status of eight Sicilian genotypes, registered into the National Cultivar Register of fruit trees in 2017 “Curcia,” “Nociara Collica,” “Panottara Collica,” “Panottara Galati Grande,” “Parrinara,” “Panottara Baratta Piccola,” “Enzo,” and “Rossa Galvagno.”

As a first step, the presence of *apple mosaic virus* (ApMV) was investigated in the donor plant material available for propagation. The virus belongs to the genus *Ilarvirus*, Bromoviridae family, and is able to infect 19 different families, including Betulaceae (hazelnut) and Rosaceae (over 65 species) (Gotlieb and Berbee, 1973; Wong and Horst, 1993; Aramburu and Rovira, 1998; Tzanetakis and Martin, 2005; Lakshmi et al., 2011). In some hazelnut cultivars the virus causes the reduction of both nut size and yield (Kobylko et al., 2005). Yield losses can reach 42% and are associated with leaf symptoms such as chlorotic or yellow areas, rings, and mosaic patterns (Cieślińska and Valasevich, 2016). In the cultivar Negret healthy trees yielded 77% higher than the infected ones (Aramburu and Rovira, 1998).

The traditional nursery propagation method of hazelnuts is based on the use of suckers from mound layering, a time-consuming technique with low efficiency at the commercial level (Mardani et al., 2020). Therefore, the micropropagation method is not only considered as an appropriate alternative for producing high quality, disease-free, and true-to-type hazelnut plants, independent from the season and environmental conditions (Debergh et al., 1991), but also it represents a valuable tool for plant biodiversity conservation (Sgueglia et al., 2018, 2019). However, hazelnut genotypes commonly show a difficult *in vitro* establishment due to surface sterilization failure, strictly related to the very common problem of endogenous contamination, showing even after weeks or months after culture initiation, besides to the lack of young plant material (Hand et al., 2016; Javed et al., 2017). Thus, a successful micropropagation protocol is highly dependent on the efficacy of surface sterilization and benefits from the use of the first three nodes below the apex of rapidly developed greenhouse plants (Hand et al., 2016). Encapsulation technology proved to be a powerful multiplication technique which can sustain standard tissue culture protocols with high viability, regrowth, and conversion rates. Moreover, meristem tip culture (MTC) protected by the synthetic seeds technique (MTC-SS) proved to be a powerful sanitation technique in eliminating various viruses associated with the Fig mosaic disease (FMD) (Yahyaoui et al., 2017). Therefore, an efficient and valid phytosanitary certification program is highly necessary in Sicily to provide growers with superior elite selected genotype of hazelnut (Nebrodi mountains), well adapted to the Sicilian environmental conditions. The main objective of this work is to provide healthy and true-to-type Sicilian hazelnut genotypes, *via* the *in vitro* sanitation technique using encapsulated meristem tips previously successfully adopted for *Ficus carica* L. virus elimination (Yahyaoui et al., 2018).

## Materials and Methods

### Plant Material

Young suckers of eight genotypes of hazelnut, namely “Curcia,” “Nociara Collica,” “Panottara Collica,” “Panottara Galati Grande,” “Parrinara,” “Enzo,” “Rossa Galvagno,” and “Panottara Baratta Piccola,” were selected among the eleven hazelnut genotypes located in the Nebrodi area (Sicily, Italy, Farms in municipality of Naso, Tortorici, Ucria, San Piero Patti) and successfully cultivated in pots at the SAAF greenhouse. The *in vitro* cultures were carried out at the Department of Agricultural, Food, and Forest Sciences (SAAF) of the University of Palermo.

### Extraction of Total Nucleic Acids and Reverse Transcriptase-PCR for *Apple Mosaic Virus* Screening

Reverse transcriptase (RT)-PCR tests were made on total nucleic acids (TNAs) extracted from bark scraps of young branches since they showed to give better results in terms of RNA extract quality and quantity if compared with leaf tissues (Ertunc et al., 2014) and using the silica capture method as described by Foissac et al. (2001). Bark scraps were frozen in liquid nitrogen and ground using mortar and pestle; the obtained powder was macerated with 1 mL of buffer (6 M guanidine thiocyanate containing 0.2 M sodium acetate, 25 mM EDTA, 1 M potassium acetate, 2.5% PVP-40 and 1% β-mercaptoethanol). The homogenized solution was transferred into labeled tubes and 100 μL of 10% sodium lauryl sarcosyl was added. The mixture was incubated at 70°C with intermediate shaking, and then placed in ice for 5 min. Following a centrifugation at 13,000 rpm for 10 min, 300 μL of supernatant were transferred into new eppendorf tubes and 300 μL of 6 M sodium iodide, 50 μL silica, and 150 μL absolute ethanol were added. The mixture was shaken gently at room temperature for 30 min and centrifuged at 6,000 rpm for 1 min. The pellet was washed three times with 500 μL of washing buffer (10 mM Tris–HCl containing 0.05 mM EDTA, 50 mM NaCl, and 50% absolute ethanol). The pellet was dried at room temperature for 10 min, resuspended in 150 μL of RNase free water and incubated at 70°C for 4 min. Following centrifugation at 13,000 rpm for 3 min, 150 μL of the supernatant was transferred into a new eppendorf tube and stored at –20°C. One step RT-PCR was performed using the primer pair designed for the detection of ApMV by Menzel et al. (2002), since no amplification was obtained with other primer sets previously tested for ApMV (Ertunc et al., 2014). The PCR mixture contained virus-specific forward and reverse primers (0.2 μM each) (Table 1), 1.5 mM dNTPs mix (Thermofisher), 2.5 μL 10 X Taq buffer (Thermofisher), 8 U MMLV_RT (Invitrogen), 1.2 U RNase inhibitor (Promega), 0.6 μM MgCl_2_, 1 U Taq DNA Polymerase (Thermofisher), 2 μL RNA template in a final volume of 25 μL adjusted with sterile distilled water. PCR amplifications were conducted in a Bio-Rad C1000 thermal cycler. PCR cycles were as the following: 30 min at 42°C, 15 min at 95°C and 34 cycles of 30 s at 94°C, 30 s at 62°C and 1 min at 72°C; a final extension step at 72°C was carried out for 7 min. The amplified DNA fragments were electrophoresed in 1% agarose gel, visualized, and photographed under UV light. Positives genotypes were recollected, rescreened for the presence of ApMV, and maintained at SAAF greenhouse for further use.

**TABLE 1:** List of specific forward and reverse primers used in reverse transcriptase (RT)-PCR for *Apple mosaic virus* (ApMV) genome amplification (Menzel et al., 2002).

Virus name	Primers	Primers sequences (5′–3′)	Amplicon size (bp)	Ann. temp. (°C)
ApMV	Ap-s	ATC CGA GTG AAC AGT CTA TCC TCT AA		
	Ap-a	GTA ACT CAC TCG TTA TCA CGT ACA A	262	62

### DNA Typing of Cultivars

The eight previously mentioned genotypes were DNA typed by the analysis of simple sequence repeat markers (SSRs) at the Department of Agricultural, Food, and Forest Sciences (DISAFA) of the University of Torino. For each genotype, young leaves were collected from both donor plants cultivated in the open field in the Nebrodi area (Sicily, Italy) and from plants maintained at SAAF greenhouse with (16 samples in total). DNA was extracted following the procedure described by Doyle and Doyle (1987). Samples were genotyped using a set of 10 SSR loci: CaT-B107, CaT-B501, CaT-B502, CaT-B503, CaT-B504, CaT-B505, CaT-B507, CaT-B508 (Boccacci et al., 2005), CaC-B020, and CaC-B028 (Bassil et al., 2005). PCR amplifications were performed in a volume of 15 μL containing 50 ng DNA, 0.5 U Taq-DNA polymerase (Kapa Biosystems, Wilmington, MA, United States), 1.5 μL 10X PCR Buffer, 2.2 mM MgCl_2_, 200 μM dNTPs, and 0.5 μM of each primer. PCR products were analyzed on a 3,130 Genetic Analyzer (Applied Biosystems, Foster City, CA, United States) and data analysis was performed with Gene Mapper 4.0 software; alleles were defined by their size in base pairs, by comparison with the standard size (Gene Scan 500 LIZ, Applied Biosystems).

### *In vitro* Establishment

#### Sterilization

Nodal segments were collected from hazelnut plant material that was positive to ApMV and *in vitro* multiplied as an infected source stock to undergo the sanitation techniques. The shoot apex was discarded since it showed to be too sensitive to the sterilization procedures dying within few days of culturing (Bacchetta et al., 2008; Hand et al., 2016). Axillary singular buds and the entire nodal segments were collected at different sampling periods (P1 = December; P2 = January; P3 = July, and P4 = September). Both types of explants were subjected to a cold storage treatment at 4°C for 15 days and compared with those cultured directly and without being subjected to a cold treatment.

For the sterilization of the plant material, protocol by Silvestri et al. (2020) with some modifications was used briefly, explants were washed under running tap water for 2 h; immersed for 1 h into an aqueous solution containing 250 mg L^–1^ ascorbic acid, 250 mg L^–1^ citric acid, 5 mg L^–1^ GA_3_, and 0.1% of plant preservative mixture germicide (PPM); treated with 0.1% solution of mercury chloride (HgCl_2_) for 2 min and 3% solution of hydrogen peroxide (H_2_O_2_) for 3 min; finally they were treated with 70% ethanol for 10 min using vacuum under the laminar flow hood to remove all air bubbles and provide a more efficient sterilization process. Explants were further sterilized in a 35% commercial bleach solution containing few drops of Tween 20 for 20 min and rapidly rinsed twice with sterile distilled water (each for 5 min). Finally, sterilized microcuttings were dried under sterile laminar flow hood for 10 min.

#### Culture Establishment

**FIGURE 1:**
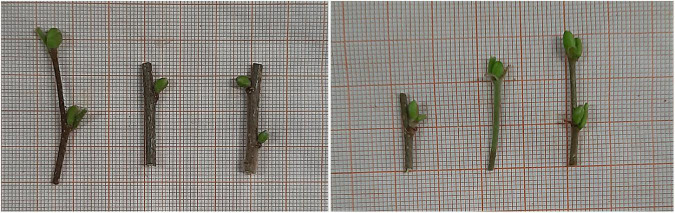
Collected cuttings with one or two buds of hazelnut “Curcia” for the sterilization procedure during July (P3).

Two types of explants were tested in this study: axillary singular buds, excised from the sterilized microcuttings, and the entire microcutting, containing one or more buds (Figure 1) were plated on a solid culture medium (N1). Damiano et al. (2005) protocol was adopted for our current study to increase the *in vitro* stock plant material for further sanitation techniques. DKW mineral salts and vitamin mixture (Driver and Kuniyuki, 1984) were used, supplemented with 27.8 mg L^–1^ FeSO_4_.7H_2_0 and 37. 3 mg L^–1^ Na_2_EDTA (Damiano et al., 2005), 30 g L^–1^ sucrose, 2 mg L^–1^ BAP, 0.1 mg L^–1^ GA_3_, 0.01 mg L^–1^ IBA, solidified with 6.5 g L^–1^ plant agar (Duchefa Biochem, Italy). After the pH adjustment to 5.7, the medium was autoclaved at 120°C for 20 min. The cultures were then sealed with Parafilm, labeled and maintained under light conditions (16/8-h light/dark photoperiod), with light supplied by cool-white fluorescent lamps (TL-D 36 W/865; Philips, Suresnes, France) at a photosynthetic photon flux density (PPFD) of 35 μmol m^–2^s^–1^. After 4 weeks from culture establishment, the number of sprouted explants was recorded and statistically analyzed. The cultures were subcultured weekly into a fresh medium. Contamination was checked with a 3-day interval. In order to develop an efficient protocol for *in vitro* hazelnut micropropagation, factorial experiments were designed to quantify interactions and to identify the optimal combination of factors with the aim of improving explants response. Therefore, we statistically analyzed the effect of different factors such as cultivar (C), sampling time (St), explant type (E), and cold treatment (T) on *in vitro* shoot proliferation. We analyzed each factor by one-way and univariate factorial ANOVA as well as their interaction as fixed factors using IBM^®^ SPSS^®^ Statistics 20 (IBM, 2011). For each factor, the mean of responding explants as proliferated shoots [six replications (Petri dishes) with five explants for each cultivar and per treatment] was expressed as a percentage ± standard deviation (±SD) relative to the total amount of cultured explants. Data analysis was followed by Tukey’s multiple comparison test at *p* ≤ 5%.

#### Standard Meristem Tip Culture and Meristem Tip Culture-Synthetic Seed Techniques

The practical applicability of MTC-SS technique was evaluated on various hazelnut cultivars. Meristem tips of about 0.2–0.3 mm in size and containing one or two primordial leaves were excised from the *in vitro* proliferated hazelnut shoots positive to the ApMV. Explants were then encapsulated and viability (explants with a green appearance, without necrosis or yellowing), regrowth (encapsulated meristem tips that produced shoots ≥ 4 mm), and conversion rate (simultaneous extrusion of shoots and roots at least 4 mm long from encapsulated microcuttings) were evaluated. Obtained results were compared to those of the traditional approach of MTC. For each cultivar, a total of six Petri dishes were used where 30 meristems were encapsulated by applying the synthetic seeds technique described by Yahyaoui et al. (2017). Five synthetic seeds were then sown in each Petri dish (60 × 15 mm), containing 10 mL of DKW medium, supplemented with 0.7 mg L^–1^ Gibberellic acid (GA_3_), 2 mg L^–1^ of 6-benzylaminopurine (BAP), 2.4 mg L^–1^ indole-3-butyric acid (IBA), and 20 g L^–1^ sucrose. The cultures were incubated in a growth chamber at (23 ± 1)°C under a 16/8-h (light/dark) and a PPFD of 40 μmol m^–2^ s^–1^. The well and successfully sprouted shoots of the tested cultivars from the MTC-SS technique were selected for the evaluation of shoot length (mm) and leaf number. Recorded data were statistically analyzed by one-way ANOVA using IBM^®^ SPSS^®^ Statistics 20 (IBM, 2011), followed by Tukey’s multiple comparison test at *p* ≤ 5%. Results are expressed as mean ± standard error (±SE).

#### Efficiency of *Apple Mosaic Virus* Eradication by Meristem Tip Culture-Synthetic Seeds

Regenerated plantlets from the encapsulated meristem tips were tested again by RT-PCR to confirm the elimination of ApMV. Plantlets were considered as virus-free when there was no product amplification by RT-PCR. The ratio number of virus-free plantlets/number of initial tested plantlets was used to calculate the efficiency of the protocol for virus-free plant production.

## Results

### Reverse Transcriptase-PCR for Assessing the Presence of *Apple Mosaic Virus*

Extracted RNAs were amplified with the Menzel et al. (2002) primers for coat protein gene of ApMV. The amplified products of RT-PCR were 262 bp long fragments. Although, some amplified RNAs showed clear bands on electrophoresis gel, tissues of the bark of young branches gave a satisfactory result for total RNA extraction and reliable amplification of ApMV in one step RT-PCR, confirming the previous findings by Ertunc et al. (2014). The eight hazelnut samples from “Curcia,” “Nociara Collica,” “Panottara Collica,” “Panottara Galati Grande,” “Parrinara”, “Panottara Baratta Piccola,” “Enzo,” and “Rossa Galvagno” genotypes resulted positive to ApMV (Supplementary Figure 1).

### DNA Typing of Cultivars

Ten SSR markers were used for the DNA-typing of the eight cultivars to determine their genetic profiles and assess their identity (Table 2). In all cases, the samples (“Curcia,” “Nociara Collica,” “Panottara Collica,” “Panottara Galati Grande,” “Parrinara,” “Panottara Baratta Piccola,” “Enzo,” and “Rossa Galvagno”) collected in the field and in greenhouse, showed the same genetic profile, confirming that the material in greenhouse used to start the propagation and the certification process was “true to type.” SSR data, compared with genetic profiles of cultivars present in the database developed by DISAFA (Boccacci et al., 2006, 2013), showed that seven out of eight genotypes have unique profiles, whereas “Curcia” resulted to have the same genotype of “Nocchione” and “Mansa;” the first is a cultivar grown in Latium, the second is a Sicilian cultivar spread in the Nebrodi area. Interestingly, the “Panottara” genotypes were different from each other and thus correctly held to be true different cultivars. Yet, “Panottara Baratta Piccola” shared the same genotype with “Panottara” the genotype selected as standard during surveys within the EU AGRI GENRES project SAFENUT (2008–10).

**TABLE 2:** Genetic profiles of the cultivars analyzed at 10 microsatellite loci (SSR); allele size is expressed in base pairs.

Locus SSR		CaT-B501		CaT-B504		CaT-B502		CaT-B107		CaC-B028		CaT-B507		CaT-B508		CaT-B503		CaT-B505		CaC-B020	
Cultivar		Alleles		Alleles		Alleles		Alleles		Alleles		Alleles		Alleles		Alleles		Alleles		Alleles	
*Curcia*	Open field	116	130	161	185	185	191	114	124	255	263	182	192	158	158	123	123	122	128	279	285
	Greenhouse	116	130	161	185	185	191	114	124	255	263	182	192	158	158	123	123	122	128	279	285
*Nociara Collica*	Open field	124	130	171	185	185	191	122	124	255	257	180	182	158	164	123	123	124	128	277	285
	Greenhouse	124	130	171	185	185	191	122	124	255	257	180	182	158	164	123	123	124	128	277	285
*Panottara Collica*	Open field	116	126	171	185	185	185	122	136	257	278	192	196	158	162	123	123	116	128	287	287
	Greenhouse	116	126	171	185	185	185	122	136	257	278	192	196	158	162	123	123	116	128	287	287
*Panottara Galati Grande*	Open field	124	130	171	185	185	185	114	122	263	278	180	192	158	158	123	123	116	128	285	287
	Greenhouse	124	130	171	185	185	185	114	122	263	278	180	192	158	158	123	123	116	128	285	287
*Parrinara*	Open field	116	130	161	185	191	199	120	124	255	269	182	192	158	168	115	123	120	122	285	285
	Greenhouse	116	130	161	185	191	199	120	124	255	269	182	192	158	168	115	123	120	122	285	285
*Panottara Baratta Piccola*	Open field	116	122	175	181	187	203	122	122	263	267	180	182	158	158	115	123	122	128	275	279
	Greenhouse	116	122	175	181	187	203	122	122	263	267	180	182	158	158	115	123	122	128	275	279
*Enzo*	Open field	126	130	173	185	191	191	120	124	255	257	190	192	158	158	123	129	120	128	285	287
	Greenhouse	126	130	173	185	191	191	120	124	255	257	190	192	158	158	123	129	120	128	285	287
*Rossa Galvagno*	Open field	124	130	171	185	185	185	114	114	257	263	190	192	158	166	123	129	116	128	285	287
	Greenhouse	124	130	171	185	185	185	114	114	257	263	190	192	158	166	123	129	116	128	285	287

### *In vitro* Culture

**FIGURE 2:**
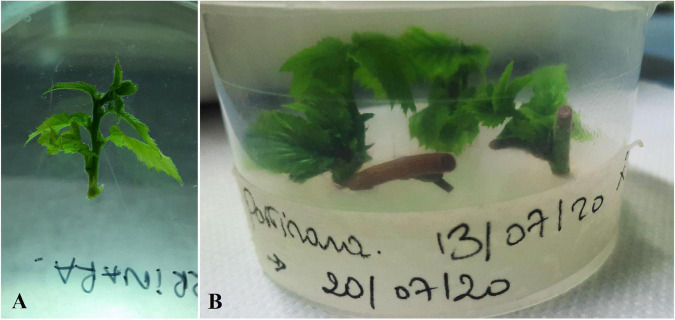
Shoot proliferation from cuttings of the variety “Parrinara,” sampled at two different times. **(A)** September (P4); **(B)** July (P3).

**FIGURE 3:**
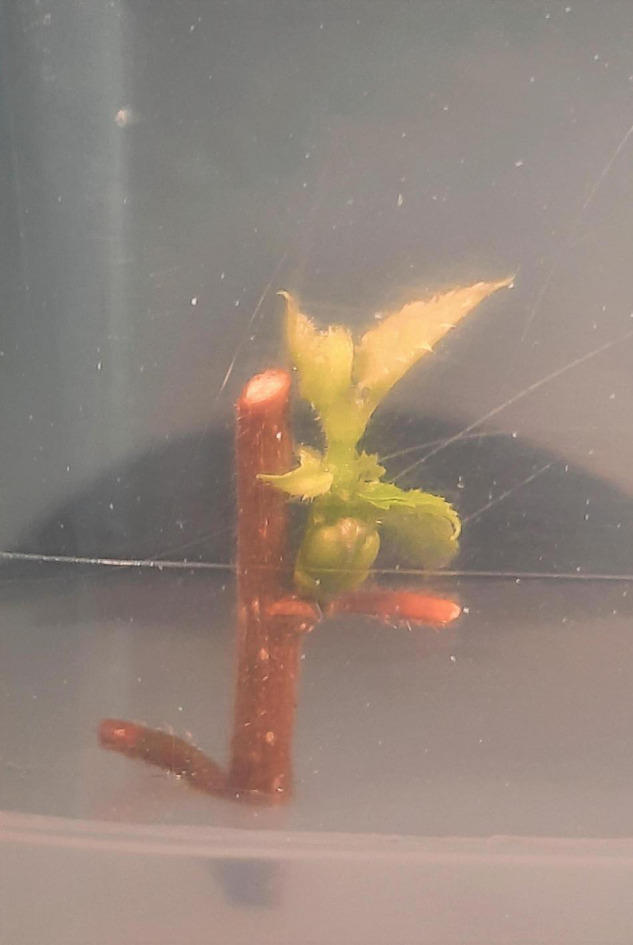
Sprouted plantlets of hazelnut “Rossa Galvagno” from cuttings sampled in September (P4) from greenhouse plants.

To develop a quick and efficient protocol for *in vitro* multiplication of *Corylus avellana* L., the effect of cultivar, sampling time, cold treatment, and the type of explant used were statistically evaluated on hazelnuts shoot proliferation rates expressed as percentage [±standard deviation (±SD)]. Results showed that all hazelnut cultivars succeeded to sprout with different frequencies except for “Panottara Baratta Piccola,” which expressed a high contamination rate even after 1 month from culture initiation and failed to be successfully established *in vitro*. A significant difference of shoot proliferation rate among the seven cultivars was observed (*p* ≤ 0.01). The variety “Parrinara” revealed the highest shoot proliferation rate with 35%, followed by “Panottara Galati Grande” (32%), “Nociara Collica” (32%) and “Rossa Galvagno” (31%) (Figures 2, 3). A moderate shoot proliferation rate was registered for “Curcia” (23%) whereas the cultivars with the lowest percentage of sprouting were “Enzo” and “Panottara Collica” (11 and 2%, respectively) (Tables 3, 4). Concerning the time for explant sampling, a highly statistically significant difference of shoot proliferation rates was observed between dates (*p* ≤ 0.01). The fourth timing (September) gave the best shoot proliferation rate (49%) followed by the third one (July) with 33%. “Parrinara,” “Panottara Galati Grande,” “Rossa Galvagno,” and “Nociara Collica” cultivars gave the highest shoot proliferation rates ranging between 43 and 66% during P3 and P4, respectively. Moreover, “Curcia” and “Enzo” cultivars showed the best shoot proliferation rates (66 and 28%, respectively) during September, while lower values were registered during July (15 and 14%, respectively) (Tables 5, 6). Explant types used in this experiment showed significant differences in terms of regenerated explants (*p* ≤ 0.01). Moreover, as shown in Table 3, the entire cultured cuttings having one or more buds gave higher regenerated explants percentage (35%), when compared to the use of single buds (12%) planted directly onto the culture media. However, no statistically significant effect was recorded for the cold storage treatment at 4°C on the shoot proliferation rates (*p* = 0.144) at 5% level. The analysis of variance showed a highly significant difference for the interaction of the factors cultivar, sampling time, and explant type used for hazelnut micropropagation (*p* = 0.001). It is important to report also that the cultivar “Panottara Collica” was very difficult to stabilize *in vitro* at the beginning, but it showed a great performance during the multiplication phase.

**TABLE 3:** Effects of cultivars, time of sampling, cold treatment and type of explants on *Corylus avellana* L. shoot proliferation rates (SD ± standard deviation).

Cultivars (C)						
	Total number (*N*)	Mean	±SD	95% Confidence interval for mean		*P* value
				Lower bound	Upper bound	
*Curcia*	480	23	41.8	19	26	≤0.01*
*Nociara Collica*	480	32	46.6	28	36	
*Panottara Collica*	480	2	13.6	1	3	
*Panottara Galati Grande*	480	32	46.7	28	36	
*Parrinara*	480	35	47.9	31	4	
*Enzo*	480	11	30.8	8	13	
*Rossa Galvagno*	480	31	46.3	27	35	
Total	3360	24	42.5	22	25	
**Sampling time (St)**						
December (**P1**)	840	4	19.7	3	5	≤0.01*
January (**P2**)	840	8	27.8	7	10	
July (**P3**)	840	33	47.1	3	36	
September (**P4**)	840	49	50.0	45	52	
Total	3360	24	42.5	22	25	
**Explant type (E)**						
Singular buds	1680	12	32.5	10	14	≤0.01*
Cuttings with one or two buds	1680	35	47.8	33	38	
Total	3360	24	42.5	22	25	
**Treatment (T)**						
Without cold treatment at 4°C	1680	23	41.8	21	25	0.144
With cold treatment at 4°C	1680	25	43.1	23	27	
Total	3360	24	42.5	22	25	
**Interactions: tests of between-subjects effects**						
C × St						0.007*
C × E						0.076
C × T						0.798
St × E						≤0.01*
St × T						0.152
E × T						0.344
C × St × E						≤0.01*
C × St × T						0.011*
C × E × T						0.001*
St × E × T						0.001*
C × St × E × T						1.000

*The mean difference is significant at the 0.05 level.

**TABLE 4:** Tukey’s *post hoc* tests for multiple comparisons.

Cluster of cultivars		Mean difference (I–J)	±SE	*P* value	95% Confidence interval	
					Lower bound	Upper bound
*Curcia*	*Nociara Collica*	–9.4*	2.6	0.007*	–17	–2.0*
	*Panottara Collica*	20.6*	2.6	≤0.01*	13	28*
	*Panottara Galati Grande*	–9.6*	2.6	0.005*	–17	–2.0*
	*Parrinara*	–12.9*	2.6	≤0.01*	–21	–5.0*
	*Enzo*	11.9*	2.6	≤0.01*	4.0	20*
	*Rossa Galvagno*	–8.5*	2.6	0.021*	–16	–1.0*
*Nociara Collica*	*Curcia*	9.4*	2.6	0.007*	2.0	17*
	*Panottara Collica*	30.0*	2.6	≤0.01*	22	38*
	*Panottara Galati Grande*	–0.2	2.6	1.00	–8.0	8.0
	*Parrinara*	–3.5	2.6	0.831	–11	4.0
	*Enzo*	21.2*	2.6	≤0.01*	13	29*
	*Rossa Galvagno*	0.8	2.6	1.00	–7.0	9.0
*Panottara Collica*	*Curcia*	–20.6*	2.6	≤0.01*	–28	–13*
	*Nociara Collica*	–30.0*	2.6	≤0.01*	–38	–22*
	*Panottara Galati Grande*	–30.2*	2.6	≤0.01*	–38	–22*
	*Parrinara*	–33.5*	2.6	≤0.01*	–41	–26*
	*Enzo*	–8.8*	2.6	0.016*	–17	–1.0*
	*Rossa Galvagno*	–29.2*	2.6	≤0.01*	–37	–21*
*Panottara Galati Grande*	*Curcia*	9.6*	2.6	0.005*	2.0	17*
	*Nociara Collica*	0.2	2.6	1.00	–8.0	8.0
	*Panottara Collica*	30.2*	2.6	≤0.01*	22	38*
	*Parrinara*	–3.3	2.6	0.868	–11	4.0
	*Enzo*	21.5*	2.6	≤0.01*	14	29*
	*Rossa Galvagno*	1.0	2.6	1.00	–7.0	9.0
*Parrinara*	*Curcia*	12.9*	2.6	≤0.01*	5.0	21*
	*Nociara Collica*	3.5	2.6	0.831	–4.0	11
	*Panottara Collica*	33.5*	2.6	≤0.01*	26	41*
	*Panottara Galati Grande*	3.3*	2.6	0.868	–4.0	11*
	*Enzo*	24.8*	2.6	≤0.01*	17	33*
	*Rossa Galvagno*	4.4*	2.6	0.643	–3.0	12*
*Enzo*	*Curcia*	–11.9*	2.6	≤0.01*	–20	–4.0*
	*Nociara Collica*	–21.2*	2.6	≤0.01*	–29	–13*
	*Panottara Collica*	8.8*	2.6	0.016*	1.0	17*
	*Panottara Galati Grande*	–21.5*	2.6	≤0.01*	–29	–14*
	*Parrinara*	–24.8*	2.6	≤0.01*	–33	–17*
	*Rossa Galvagno*	–20.4*	2.6	≤0.01*	–28	–13*
*Rossa Galvagno*	*Curcia*	8.5*	2.6	0.021*	1.0	16*
	*Nociara Collica*	–0.8*	2.6	1.00	–9.0	7.0*
	*Panottara Collica*	29.2*	2.6	≤0.01*	21	37*
	*Panottara Galati Grande*	–1.0*	2.6	1.00	–9.0	7.0*
	*Parrinara*	–4.4*	2.6	0.643	–12	3.0*
	*Enzo*	20.4*	2.6	≤0.01*	13	28*

*The mean difference is significant at the 0.05 level.

**TABLE 5:** Recorded shoot proliferation rates (means ± SD) of seven Sicilian cultivars of *Corylus avellana* L. at different times of explant sampling.

Cultivars	Sampling time				Average shoot proliferation rates (%)
	December (P1)	January (P2)	July (P3)	September (P4)	
*Curcia*	0.0	9.0 ± 29.0	15 ± 35.9	66 ± 47.6	23 ± 41.8
*Nociara Collica*	4.0 ± 20.1	13 ± 34.1	52 ± 50.1	58 ± 49.6	32 ± 46.6
*Panottara Collica*	3.0 ± 15.7	5.0 ± 21.9	0.0	0.0	2.0 ± 13.6
*Panottara Galati Grande*	3.0 ± 18.0	11 ± 31.2	50 ± 50.2	64 ± 48.2	32 ± 46.7
*Parrinara*	5.0 ± 21.9	13 ± 34.1	58 ± 49.6	66 ± 47.6	35 ± 47.9
*Enzo*	0.0	0.0	14 ± 35.0	28 ± 45.3	11 ± 30.8
*Rossa Galvagno*	13 ± 34.1	8.0 ± 26.4	43 ± 49.8	60 ± 49.2	31 ± 46.3
Total average (%)	4.0 ± 19.7	8.0 ± 27.8	33 ± 47.1	49 ± 50.0	24 ± 42.5

**TABLE 6:** Tukey’s *post hoc* tests for multiple comparisons.

Cluster of sampling time		Mean difference (I–J)	±SE	*P* value	95% Confidence interval	
					Lower bound	Upper bound
December (**P1**)	P2	–4.4	1.9	0.087	–9.0	≤0.01
	P3	–29.2*	1.9	≤0.01*	–34	24*
	P4	–44.8*	1.9	≤0.01*	–50	–40*
January (**P2**)	P1	4.4	1.9	0.087	≤0.01	9.0
	P3	–24.8*	1.9	≤0.01*	–30	–20*
	P4	–40.4*	1.9	≤0.01*	–45	–36*
July (**P3**)	P1	29.2*	1.9	≤0.01*	24	34*
	P2	24.8*	1.9	≤0.01*	20	30*
	P4	–15.6*	1.9	≤0.01*	–20	–11*
September (**P4**)	P1	44.8*	1.9	≤0.01*	40	50*
	P2	40.4*	1.9	≤0.01*	36	45*
	P3	15.6*	1.9	≤0.01*	11	20*

*The mean difference is significant at the 0.05 level.

### Standard Meristem Tip Culture and Meristem Tip Culture-Synthetic Seed Techniques

**FIGURE 4:**
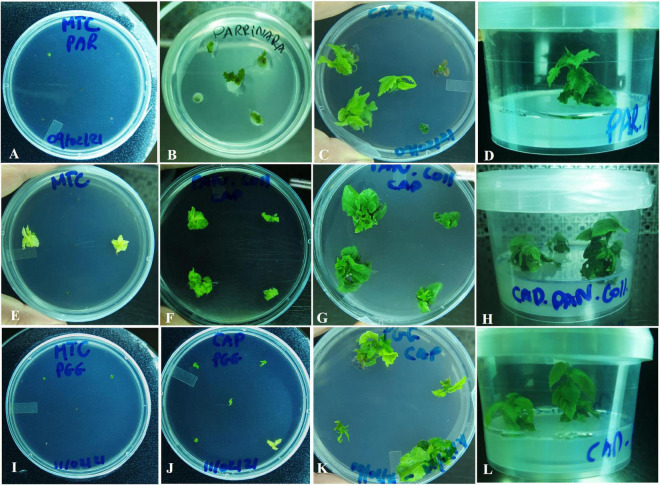
*In vitro* shoot proliferation of some Sicilian hazelnut cultivars. **(A)** Standard meristem tip culture (MTC) of the variety “Parrinara”. **(B–D)** plantlets growth from encapsulated meristem tips (0.2–0.3 mm) of the variety “Parrinara” using the encapsulation technology; **(E)** Standard MTC of the variety “Panottara Collica”; **(F–H)** plantlets growth from encapsulated meristem tips (0.2–0.3 mm) of the variety “Panottara Collica” using the encapsulation technology; **(I)** Standard MTC of the variety “Panottara Galati Grande”; **(J–L)** plantlets growth from encapsulated meristem tips (0.2–0.3 mm) of the variety “Panottara Galati Grande” genotype using the encapsulation technology.

This investigation studied the use of both conventional sanitation technique *via* direct MTC and the non-conventional one, using MTC combined with the synthetic seed technology (MTC-SS). A high and satisfactory viability (65%) and moderate to satisfactory regrowth rates (26%) were registered using the MTC-SS sanitation technique for the diseased *in vitro* stock plant material. No conversion rates were obtained for all the encapsulated cultivars that started to develop callus instead, thus they were rooted separately. “Parrinara,” “Rossa Galvagno,” and “Panottara Galati Grande” cultivars gave the highest and almost similar viability rates (93, 93, and 80%, respectively), followed by “Curcia” (60%), “Panottara Collica” (60%), and “Nociara Collica” (47%), whereas “Enzo” harbored the lowest viability rate with 23%. “Parrinara” showed the best regrowth rate (53%), followed by “Panottara Galati Grande” (30%), “Panottara Collica” (30%), “Curcia” (23%), “Rossa Galvagno” (23%), and “Nociara Collica” (13%). Rooting of microcuttings in one step was not achieved; therefore obtained plantlets were multiplied and rooted separately for future investigations. On the other hand, the obtained viability (7%) and regrowth (2%) rates using the standard MTC were too low for all the experimented genotypes with a statistically significant effect between cultivars at 5% level (*p* = 0.004; Table 7 and Figures 4A,E,I). A high *in vitro* performance was observed for “Panottara Collica,” “Panottara Galati Grande,” and “Parrinara” cultivars, in terms of growth and shoot proliferation from the MTC-SS technique if compared with the standard MTC approach (Figure 4). Therefore, these cultivars were selected for shoot length (mm) and leaf number evaluation. Statistical analysis revealed a highly significant difference for shoot length among the regenerated presanitized plantlets obtained after 16 weeks of culture initiation (*P* = 0.003). These values were high and comparable for the three cultivars and ranged from 14.7 mm for “Parrinara” to 11.6 mm for “Panottara Galati Grande” with a total mean shoot length of 13.4 mm. In addition, no significant difference of leaf number per explant was observed. The number of leaves per shoot was high and comparable for the three evaluated cultivars (mean: 8.7), where “Parrinara” harbored the best value (10), followed by “Panottara Galati Grande” (8.4), and “Panottara Collica” (Table 8).

**TABLE 7:** Viability and regrowth responses (means ± SD) to both conventional (MTC) and non-conventional (MTC-SS) multiplication techniques in seven cultivars of *Corylus avellana* L. synthetic seeds, after 12 weeks from sowing.

*Cultivars*	*MTC*		*MTC-SS*	
	*Viability* (*%*)	*Regrowth* (*%*)	*Viability* (*%*)	*Regrowth* (*%*)
*Curcia*	3.0 ± 18.3	3.0 ± 18.3	60.0 ± 49.8	23.0 ± 43
*Nociara Collica*	7.0 ± 25.4	3.0 ± 18.3	47.0 ± 50.7	13.0 ± 34.6
*Panottara Collica*	7.0 ± 25.4	0.00	60.0 ± 49.8	30.0 ± 46.6
*Panottara Galati Grande*	10.0 ± 30.5	3.0 ± 18.3	80.0 ± 40.7	30.0 ± 46.6
*Parrinara*	10.0 ± 30.5	7 ± 25.4	93.0 ± 25.4	53.0 ± 50.7
*Enzo*	0.00	0.00	23.0 ± 43	10.0 ± 30.5
*Rossa Galvagno*	10.0 ± 30.5	0.00	93.0 ± 25.4	23.0 ± 43
Tot	7.0 ± 25.0	2.0 ± 15.3	65.0 ± 47.7	26.0 ± 44.1
Significance*	0.004*	0.003*	≤0.05*	≤0.05*

*The mean difference is significant at the 0.05 level.

**TABLE 8:** Shoot length and number of leaves (means ± SE) of encapsulated seeds of cultivars “Panottara Collica,” “Panottara Galati Grande” and “Parrinara” after 16 weeks.

Cultivar	Shoot length (mm)	Leave numbers (*n*)
*Panottara Collica*	13.98 ± 0.14	7.67 ± 0.67
*Panottara Galati Grande*	11.57 ± 0.09	8.43 ± 0.61
*Parrinara*	14.73 ± 0.14	10.00 ± 0.63
Average	13.43 ± 0.07	8.70 ± 0.38
Significance*	0.003*	0.873

*The mean difference is significant at the 0.05 level.

### Efficiency of *Apple Mosaic Virus* Eradication by Meristem Tip Culture-Synthetic Seeds

**FIGURE 5:**
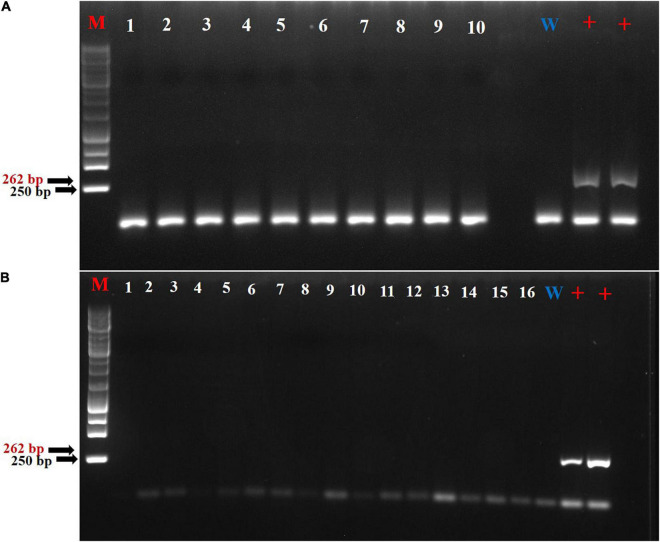
Agarose gel electrophoresis of reverse transcriptase (RT)-PCR products of sanitized plantlets subjected to the meristem tip culture-synthetic seeds (MTC-SS) technique for *Apple mosaic virus* (ApMV) screening using specific primers for a 262 bp region of the virus. **(A)** Lane M: Molecular marker 250 bp; 1–10: “Panottara Collica” cultivar isolates; W, water; +, positive control; **(B)** Lane M: Molecular marker 250 bp; 1–16: “Parrinara” cultivar isolates; W, water; +, positive control.

The effectiveness of MTC-SS sanitation technique was evaluated verifying the ApMV elimination from the plantlets. The preliminary results showed that the encapsulated excised meristems, obtained from *in vitro* multiplied plantlets and previously positively tested for ApMV, produced well-formed shoots and had no symptomatic signs of infection. The total registered average of virus elimination efficiency was 96.9% and almost similar for all tested cultivars. RT-PCR molecular analysis revealed that ApMV was totally eradicated with 100% of elimination rate from “Nociara Collica,” “Panottara Collica,” “Parrinara,” and “Enzo” cultivars followed by “Curcia” (94.12%), “Rossa Galvagno,” (93.33%) and “Panottara Galati Grande” (90%), respectively (Figure 5 and Table 9).

**TABLE 9:** *Apple mosaic virus* (ApMV) sanitary status of regenerated hazelnut plantlets subjected to the MTC-SS technique.

Cultivars	Curcia	Nociara Collica	Panottara Collica	Panottara Galati Grande	Parrinara	Enzo	Rossa Galvagno	TOT
*Virus-free plantlets/tested plantlets*	*16/17*	*16/16*	*23/23*	*18/20*	*27/27*	*10/10*	*14/15*	*124/128*
*Proportion of virus-free plantlets*	**94.12%**	**100%**	**100%**	**90%**	**100%**	**100%**	**93.33%**	**96.90%**

*Bold ones are percentage values, italic ones are absolute values.*

## Discussion

### Reverse Transcriptase-PCR for Detection of *Apple Mosaic Virus*

The obtained results confirmed the detection of the most economically damaging *Ilarvirus* affecting hazelnut by one step RT-PCR applied on a set of local Sicilian cultivars using dormant tissues, as described by Ertunc et al. (2014), overcoming the problem of having high amount of phenolic compounds, tannins in particular. The latter are considered a problem for the extraction of good quality RNA by several researchers that tried the herbaceous host inoculation (Sokmen, 2003) and flower tissues extraction (Akbas and Degirmenci, 2010). Moreover, this study provided further information on the sanitary status and ApMV incidence within eight Italian hazelnut genotypes showing the presence of the virus in all of them. These results are not surprising considering the vegetative propagation usually applied *via* rooted suckers or cuttings used in agriculture for this species; this favors the transmission of the viral agents affecting hazelnut.

### DNA Typing of Cultivars

The results clearly proved that microsatellite DNA markers can be an effective tool for genetic identification of genotypes included in the National Cultivar Register to produce certified material. In all cases, it was possible to verify the identity of the Sicilian genotypes in the field and in greenhouse. When the SSR data were compared with the genetic database developed by DISAFA, “Curcia” resulted very likely to be a clone of “Mansa,” the most common and widespread cultivar in the Nebrodi area. The first certain evidence of hazelnut specialized cultivation in Italy could be found in some contracts between settlers and owners in Campania, dating back to the late Middle Ages (800–900 AD) and in some other documents dating back to the Norman domination in Campania (about 1030 AD). Some hazelnut grove planting rules were indicated, such as the plant spacing (10 steps between one plant and another) besides the propagation methods (Trotter, 1921). Moreover, documents dated between 1160 and 1223 AD reported the existence of irrigated hazelnut cultivation in Sicily, in particular in the area of Piazza Armerina [Alberghina, 2002, taken from Alfonso (1886)]. Regarding the origin of the crop in Sicily, Trotter (1921) excluded its introduction by Arabs and admitted its entry from Campania at the time of the Romans.

The biodiversity present on the island is documented and described in various publications (Alfonso, 1886; Ruggieri, 1949; Damigella and Alberghina, 1970, 1983; Alberghina and Damigella, 1979). However, the cultivars currently used, refer to a single prevalent genotype known with different varietal names, including “Mansa,” “Santa Maria di Gesù,” “Comune,” “Nostrale,” “Curcia,” and “Racinante” (Alberghina, 1982). Based on the genetic analyses, these cultivars cannot be distinguished from the cultivar “Nocchione” grown in Lazio (Boccacci et al., 2006, 2013), the principal pollinizer of “Tonda Gentile Romana.” Among the other varieties of minor importance, we can mention “Carrello” (Crescimanno, 1957), “Ghirara,” the group of “Minnulare,” characterized by their long-shaped fruits and late ripening period, and “Panottara” (Damigella and Alberghina, 1983). In this study, we confirmed that “Panottara Baratta Piccola” was the same of the reference “Panottara” collected in Sicily in 2008–2010, during the EU AGRI GEN RES project SAFENUT (“Safeguard of almond and hazelnut genetic resources: from traditional uses to modern agro-industrial opportunities”), aimed to increase knowledge on the genetic diversity in the European hazelnut (Boccacci et al., 2013; Bacchetta et al., 2015). These results emphasize the need for a genetic identification of the hazelnut genotypes for authoritative certification and successful commercial management of elite genotypes.

### *In vitro* Micropropagation

The adopted sterilization protocol gave good results for the *in vitro* establishment of the studied local cultivars and allowed to overcome the endogenous contamination problem with different efficiency. The outcomes of this study give an insight on the appropriate sampling time and explant type to use for the *in vitro* establishment of hazelnut, and provide information on the shoot proliferation rates which showed to be highly dependent on the cultivar. Both sampling times during July and September were the best times for *in vitro* establishment of hazelnuts. Moreover, “Parrinara,” “Panottara Galati Grande,” “Nociara Collica,” and “Rossa Galvagno” harbored the highest aptitude for the *in vitro* multiplication, revealing high shoot proliferation rates and representing the best candidates for subsequent sanitation, whereas “Curcia” followed by “Panottara Collica” and “Enzo” registered the lowest shoot proliferation rates. Although these results have to be further investigated, this study gives an overview on the existing biodiversity of hazelnut genotypes in the Nebrodi area and could serve as a valuable tool easily transferred into the certification programs in Sicily. The protocol reported here provides methods for a rapid ApMV screening, *in vitro* sanitation, and massive micropropagation of elite hazelnut genotypes selected in Nebrodi context, such as “Parrinara” which presented the best carpological traits (data not shown) and a good *in vitro* response. The evaluation of sensory, biochemical, and nutritional traits is needed to provide additional information on the current selected cultivars and address growers to use the most suitable hazelnut genotypes, with the highest processing value for confectionery use and table consumption in Italy.

### *In vitro* Sanitation

Results of this research clearly showed that the combination of MTC (0.2–0.3 mm in size) technique with the encapsulation technology was more effective compared with the MTC used alone in promoting higher shoot proliferation rates of plantlets with higher mean shoot length and leaf number per shoot overcoming the survival problem, the low rates, and the frequently encountered difficulties of plantlets shoot proliferation from this size of explants (necrosis, etc.). The protected meristem tips using the synthetic seeds techniques proved to be highly effective in eliminating ApMV, one of the most important encountered problems in the production of hazelnut in Sicily, from all tested cultivars. These results confirmed the practical applicability of this technology for both propagation and sanitation of this important fruit crop, in one step, with the production of good quality artificial seeds by improving the effectiveness of standard sanitation approaches (traditional meristem tips culture). Kaya (2021) reports that ApMV resisted the sanitation attempt *via* meristem tips of 0.3–0.5 mm in size that showed to be too large for efficient virus elimination, confirming that the best size of explants ranges from 0.2 to 0.3 mm, as previously demonstrated in various crops by Kumar et al. (2009) and Ramgareeb et al. (2010).

## Conclusion

A protocol for the production of quick, healthy, and true-to-type *Corylus avellana* L. genotypes was achieved in this study. The selected local hazelnut genotypes were successfully micropropagated and sanitized using an innovative method. The encapsulation technology showed to be a promising approach as a vegetative regeneration system and a sanitation technique at the same time. The procedure described here can be easily implemented in laboratories for the production of a large number of true-to-type hazelnut plants, thus it could be adopted in pathogen-eradication method. Moreover, apical meristem culture showed its efficiency in eliminating viruses from infected plants using very small shoot tips, which, however, decreases survival and regrowth if used alone, whereas, its combination with the encapsulation technology greatly helped overcoming the biggest limitation of this sanitation technique. Therefore, the encapsulation technology optimized the shoot proliferation rates of small excised meristems and its use could help in minimizing the genetic diversity and can be used for the exchange of plant material between public and private plant tissue culture laboratories, as well as for germplasm conservation of genotypes as propagules for long-term storage. Yet, further investigations for assessing on a larger scale the efficiency and efficacy of the MTC-SS sanitation technique together with the conservation of healthy hazelnut micropropagules at 4°C are needed before transferring the methods at commercial level. The results of this study on calcium alginate encapsulation of *in vitro*-derived microcuttings of *Corylus avellana* L., confirmed the practical applicability of this technology for the propagation of this important nut crop, with the production of good quality artificial seeds. According to our knowledge, the recovery of various Sicilian hazelnut genotypes, and also the success of multiplication and sanitation, in a single step after sowing, are reported for the first time. Yet, the rooting stage is the most sensitive step (Bacchetta et al., 2004). This phase needs to be further investigated to achieve synthetic seed conversion in one single step.

These preliminary results need a field application to avoid false negatives and to evaluate the soil survival rate, the known limitation for this technology (Jung et al., 2004). Moreover, the genetic stability of regenerated plantlets has to be ascertained by molecular analysis. Following these tests, the optimized protocol for large-scale production of good quality and healthy hazelnut plants, and also their conservation as encapsulated propagules could be adopted and applied for the successful establishment of commercial production.

## Data Availability Statement

The original contributions presented in the study are included in the article/Supplementary Material, further inquiries can be directed to the corresponding author.

## Author Contributions

EY carried out the *in vitro* culture experiments, sanitation techniques, performed the statistical analysis, molecular analysis, wrote the manuscript, processed the experimental data and performed the analysis, drafted the manuscript and designed the figures, and results interpretation. DM and RB realized the DNA typing of cultivars and wrote there part in the manuscript. PR performed the DNA typing analysis and interpretation. MG was the scientific responsible and coordinator of the two projects, devised the project and the main conceptual ideas, collected the cultivars, verified and reviewed the final version of the manuscript, encouraged and facilitated the communication among authors. All authors provided critical feedback and helped shape the research, analysis, manuscript, and approved the submitted version.

## Conflict of Interest

The authors declare that the research was conducted in the absence of any commercial or financial relationships that could be construed as a potential conflict of interest. The handling editor is currently organizing a Research Topic with one of the authors, RB.

## Publisher’s Note

All claims expressed in this article are solely those of the authors and do not necessarily represent those of their affiliated organizations, or those of the publisher, the editors and the reviewers. Any product that may be evaluated in this article, or claim that may be made by its manufacturer, is not guaranteed or endorsed by the publisher.
