# Supplementary material for: Is It Possible to Produce Certified Hazelnut Plant Material in Sicily? Identification and Recovery of Nebrodi Genetic Resources, in vitro Establishment, and Innovative Sanitation Technique From Apple Mosaic Virus
Source: Front Plant Sci. 2021 Dec 16;12:778142. doi: 10.3389/fpls.2021.778142 (PMC8716929; doi:10.3389/fpls.2021.778142)

Supplementary material Figure 1. RT-PCR amplification of Sicilian *Apple Mosaic Virus* (ApMV) hazelnut isolates with specific primers for a 262 bp region of the virus. Lane M: Molecular marker 250 bp; Lane 1: ‘Panottara Baratta Piccola’; 2: ‘Panottara Galati Grande’; 3: water; 4: ‘Parrinara’; 5: ‘Nocciara Collica’; 6: ‘Curcia’; 7: ‘Rossa Galvagno’; 8: ‘Enzo’; 9: ‘Panottara Collica’; 10: water.

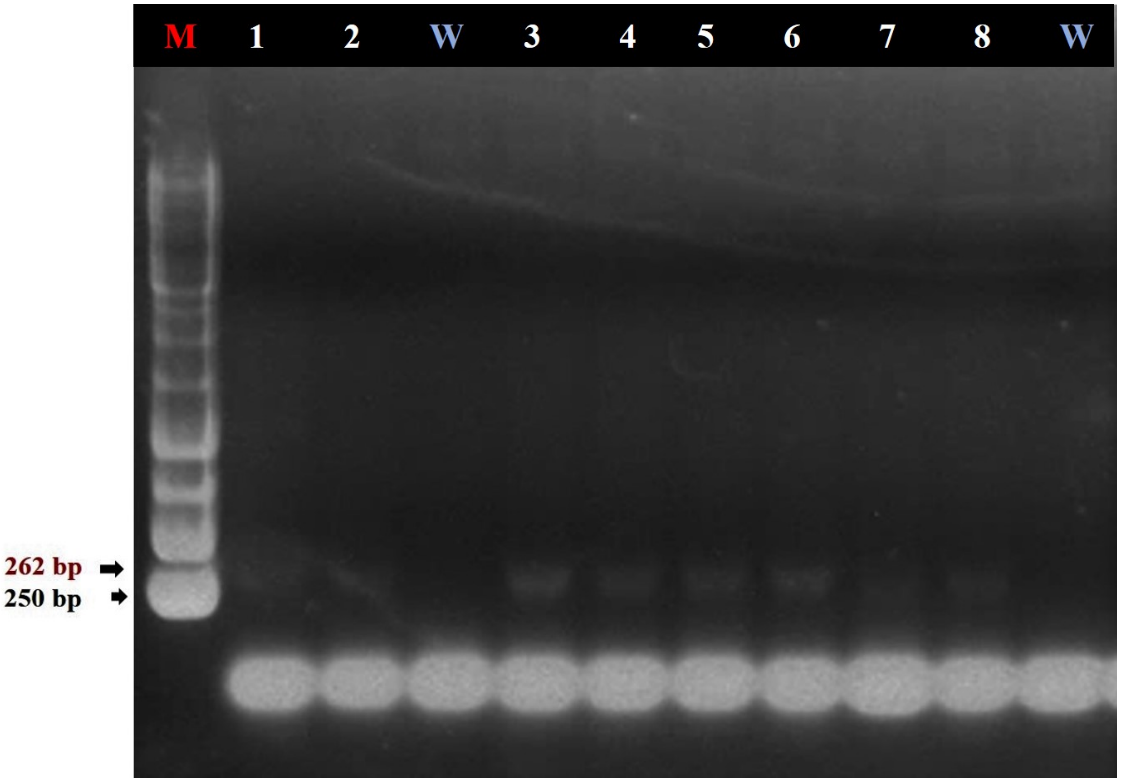

Supplement: Supplementary file 1 [file Image_1.pdf]
